# Congruency Encoding Effects on Recognition Memory: A Stage-Specific Account of Desirable Difficulty

**DOI:** 10.3389/fpsyg.2019.00858

**Published:** 2019-04-24

**Authors:** Melissa J. Ptok, Sandra J. Thomson, Karin R. Humphreys, Scott Watter

**Affiliations:** ^1^ McMaster University, Hamilton, ON, Canada; ^2^ St. Thomas University, Fredericton, NB, Canada

**Keywords:** cognitive control, memory, attention, priming, congruency, desirable difficulty

## Abstract

Recent research suggests that selectively attending to relevant stimuli while having to ignore or resist conflicting stimuli can lead to improvements in learning. While mostly discussed within a broader “desirable difficulty” framework in the memory and education literatures, some recent work has focused on more mechanistic questions of how processing conflict (e.g., from incongruent primes) might elicit increased attention and control, producing enhanced incidental encoding of high-conflict stimuli. This encoding benefit for high-control-demand or high-difficulty situations has been broadly conceptualized as a task-general property, with no strong prediction of what particular task elements should produce this effect. From stage processing models of single- and dual-task performance, we propose that memory-enhancing difficulty manipulations should strongly depend on inducing additional cognitive control at particular processing stages. Over six experiments, we show that a memory benefit is produced when increased cognitive control (*via* incongruency priming) focuses additional processing on the core meaning of to-be-tested stimuli at the semantic categorization stage. In contrast, incongruency priming targeted at response selection within the same task produces similar effects on initial task performance, but gives no memory benefit for high-conflict trials. We suggest that a simple model of limited-capacity and stage-specific cognitive control allocation can account for and predict where and when conflict/difficulty encoding benefits will occur, and may serve as a model for desirable difficulty effects more broadly.

## Introduction

The ability to deliberately focus one’s attention while ignoring irrelevant distractions has become a foundational way of defining selective attention and cognitive control (e.g., Stroop, 1935; Simon and Small, 1969; Eriksen and Eriksen, 1974). More recently, evidence suggests that selectively attending to relevant stimuli while having to ignore or resist conflicting stimuli can lead to improvements in learning (e.g., Botvinick, 2007; Verguts and Notebaert, 2008; Krebs et al., 2015; Rosner et al., 2015a). This apparent stimulus encoding benefit under high-conflict incongruent priming conditions has been typically interpreted as a task-general effect of increased cognitive control under high-conflict conditions, following the influential conflict monitoring and cognitive control model of Botvinick et al. (2001).

The present study sought to test whether these memory benefits from incongruent stimulus priming are indeed produced by this kind of task-general elicitation of greater cognitive control, or whether this incongruency memory benefit may depend on a more processing stage-specific mechanism. To anticipate our results, we show that priming with incongruent semantic information in a range of semantic categorization tasks leads to later benefits in recognition memory, but that incongruent response priming or incongruent semantic priming in more evaluative or demanding tasks does not. We argue that the locus of selective attention and cognitive control demands relative to the stage-specific processing conflict within particular task settings is a critical aspect of these differential memory effects, and show how this kind of model makes straightforward predictions about when different kinds of incongruency conflict should help or hinder later memory. We suggest that these ideas might also serve as a valuable general model for more mechanistically predicting and accounting for so-called desirable difficulty effects.

### Incongruency Effects on Memory

Congruency priming manipulations are commonly used to investigate selective attention and cognitive control. The conflict-monitoring model of cognitive control (Botvinick et al., 2001) has been an influential framework in understanding how cognitive control processes modulate selective attention in order to protect intended performance from varying degrees of interference from irrelevant stimulus information. For example, in a typical Stroop task, the conflict detected between font color and word name of an incongruent Stroop trial triggers a top-down increase in attention toward task-relevant information (in this case, the color), reducing interference from task-irrelevant information (the word name). The effects of this kind of increased cognitive control or divided attention demand on later memory have typically been seen to be negative (e.g., Craik et al., 1996; Gaspelin et al., 2013). However, several recent studies have shown that in similar kinds of congruency priming tasks, subsequent memory for incongruent (higher conflict) items can be better relative to congruent (lower conflict) items (e.g., Krebs et al., 2015; Rosner et al., 2015a). These authors have argued that the increase in selective attention due to increased cognitive control elicitation from incongruent stimuli likely provides a memory encoding benefit for incongruent items in these cases.

Krebs et al. (2015) examined the effect of “conflict”-induced cognitive control on memory using a face-word Stroop task. Participants were shown male and female faces superimposed with the word “male,” “female,” or a neutral word, and their task was to identify the gender of the face as quickly as possible. This task produced a congruency effect, where responses were fastest to congruent face-word pairs and slowest to incongruent pairs. Later, participants completed a recognition memory test for the faces, where Krebs et al. found that faces from incongruent trials were better recognized than faces from congruent or neutral trials (which did not differ). Although incongruent words interfered with the processing of face targets during the face-word Stroop task, Krebs et al. argued that top-down attentional enhancement for target information led to better incidental encoding of face stimuli in this more demanding incongruent condition.

Similar evidence for increased selective attention demands producing a recognition memory benefit comes from a series of experiments conducted by Rosner et al. (2015a). The stimuli were two interleaved words, one in red and one in green, with participants instructed to read the red word aloud. On half of the trials, the green distractor word was identical to the to-be-named red word (congruent trials), and on the other half of the trials, it was a different word (incongruent trials). Consistent with Krebs et al. (2015), they demonstrated a congruency effect in word naming, where word reading on incongruent trials was slower than on congruent trials. Incongruent words subsequently showed better recognition memory. Follow-up studies showed that this recognition memory effect was not simply driven by the additional time on task for incongruent trials in the word naming phase (Rosner and Milliken, 2015), but appears to be a consequence of the increased selective attention demands for incongruent items.

Additionally, Chiu and Egner (2015) shed new light on control processes of response inhibition. In a series of experiments, participants performed go/no-go, stop-signal, and yes/no tasks on male and female faces. Subsequent memory tests revealed that the control demands of response inhibition divert attention away from stimulus encoding, resulting in lower memory for trials with response inhibition. The negative effects of response inhibition on memory are opposite to the enhancement in memory performance when detecting and resolving conflict found in related research (Krebs et al., 2015; Rosner et al., 2015a). They suggest the conflict resolution leading to better incidental encoding in these tasks involves top-down attention toward target stimuli (Botvinick et al., 2001; Egner and Hirsch, 2005), whereas the response inhibition in their tasks directs attention away from concurrent stimulus encoding.

Similar recent research has also examined the effect of stimulus repetition on recognition memory (Collins et al., 2018; Rosner et al., 2018). In these experiments, participants underwent a study phase where they had to name a word aloud that was preceded by the same word (repeated trials) or a different word (not-repeated trials). Across experiments, these authors found that repeated words had faster reaction times (RTs) at study than non-repeated words. In a subsequent test phase, participants were presented with words and were asked to indicate whether they had seen them in the first phase of the experiment using old/new judgments. They found that non-repeated words were better remembered than repeated words. This effect remained even when repetitions at study were separated by an unrelated word and was eliminated if attention was directed toward primes. These findings provide additional evidence that attentional allocation at study may have an important impact on incidental memory encoding.

### The Present Study

In the present study, we investigated cognitive control demands induced selectively at different processing stages, and whether this stage-specific focus of control demands would influence later memory performance for high- versus low-conflict trials – that is, whether these incidental encoding effects are task-general or could be predictably stage dependent. In a typical divided attention task, cognitive control demands limit attention to information in a primary task by requiring participants to also monitor and perform a secondary task. As Chiu and Egner (2015) suggest from their recent cognitive control manipulations that direct attention away from stimulus encoding (inhibitory control tasks) rather than toward it, we suggest that in order for some difficulty manipulation (e.g., incongruent versus congruent stimulus priming) to induce a memory benefit, the difficulty must elicit increased selective attention to the information that will be later tested for a potential memory effect.

In these high- versus low-conflict/congruency situations, we suggest that the particular stage of processing that is the recipient of facilitation or conflict is a critical consideration for predicting whether a beneficial effect on later memory will occur, rather than just thinking of incongruency or conflict as eliciting greater cognitive control or attentional focus for the whole task in general. Therefore, we predict that memory enhancement effects from various encoding difficulty manipulations are not task-general effects of attention, but instead should reflect enhancement of encoding *via* cognitive control demands that do not divert the focus of this control away from the core semantic representation of task stimuli. If task difficulty in general improves incidental encoding, then we should observe a memory benefit for items encountered in more difficult task conditions, independent of which particular processing stage is involved with this conflict. Alternatively, a stage-specific account would predict that memory facilitation should only occur when an encoding difficulty manipulation enhances selective attention toward important and relevant features or the meaning of to-be-tested target stimuli.

**Figure 1:**
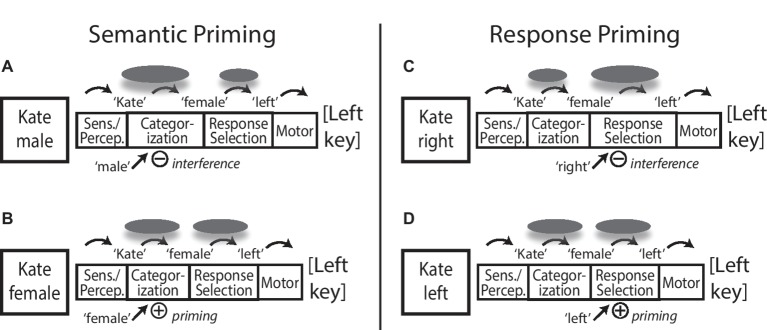
Information processing model of stage-specific incongruency encoding effects. A male/female name classification task is shown with different kinds of **(A)** incongruent versus **(B)** congruent priming of semantic categorization information, and **(C)** incongruent versus **(D)** congruent priming of response selection information. Gray ovals represent central focus of selective attention and cognitive control processes, with greater size representing proportionately greater focus and investment of processing at a given stage. Incongruent prime information induces additional cognitive control focus to the relevant information processing stage. Increased control and processing focus on semantic representations in **(A)** versus **(B)** predicts better subsequent memory for incongruently primed stimuli. Increased control and processing focus on response selection in **(C)** versus **(D)** diverts cognitive control focus away from central representation of to-be-tested stimulus information, predicting no benefit of increased conflict/control demand on later memory with incongruent response priming, despite greater attentional control and focus on the task in general. See text for more detail. Sens. = Sensation; Percep. = Perception.

Figure 1 shows examples of several theoretical situations and predictions for different kinds of congruency priming, where participants are asked to classify typical female or male names as Female or Male, responding with left or right key presses respectively (e.g., “Kate” is a female name, press the left key). The left half of the figure shows examples of semantic priming where (Figure 1A) incongruent (“male”) and (Figure 1B) congruent (“female”) distractor stimuli with task-relevant semantic feature information are shown along with the primary stimulus. Greater conflict and interference with an incongruent prime in Figure 1A elicit greater high-level attentional focus and cognitive control work (gray ovals) to resolve the classification outcome, leading to slower task RT but also more substantial attentional focus and processing of the core semantic and associative information for the stimulus name compared to the congruent condition in Figure 1B, predicting better memory for incongruently primed stimuli.

In contrast, the right half of Figure 1 shows examples of response priming where (Figure 1C) incongruent (“right”) and (Figure 1D) congruent (“left”) distractor stimuli carry task-relevant response feature information (and not semantic category information) along with the primary task stimulus. Greater conflict and interference with an incongruent response prime in Figure 1C elicit greater high-level attentional focus and cognitive control work to resolve the response selection outcome, leading to slower task RT. However, because this difference in cognitive control focus is directed away from processing and representation of the stimulus information that will later be the focus of a memory test, we predict that this situation should not lead to any memory benefit for stimuli in high-response conflict/incongruent trials.

Within this framework, we might predict that with a sufficiently strong conflict control demand at response selection, semantic or associative processing of stimulus information could in a sense be cut short, disrupting incidental encoding of stimulus information compared to the congruent condition in Figure 1D. This could lead to worse memory for incongruently primed stimuli, typical of the usual costs of divided attention and distraction, despite the overall increased attentional and cognitive control investment for the incongruent trial. In a less severe or less disruptive case, incidental encoding of stimulus information might be simply unaffected by cognitive control demands at response selection, and in that case, we would predict no congruency/difficulty differences on later memory despite processing conflict costs on initial RT performance. Importantly, in both cases, there is a strong prediction that there should not be a later memory benefit of task incongruency/conflict difficulty when that difficulty diverts the focus of cognitive control away from the representation of stimulus information. In this sense, we propose that so-called “desirable” difficulty for future memory benefit is not a task-general property, but needs to be considered as a processing stage-specific effect where the stimulus information that will be the focus of a later memory test needs to be the focus of conflict-elicited cognitive control focus.

We conducted six experiments, where we used these kinds of congruency/interference priming manipulations to selectively influence different stages of task processing, and then assessed the influence of these stage-specific manipulations on later recognition memory for initial task stimuli. Experiments 1 and 2 used response congruency/priming to target response selection, independent of semantic information for to-be-tested stimuli. Experiments 3 through 6 used a range of different tasks with semantic congruency/priming to assess the generalizability of a semantic focus explanation for these effects, and to assess potential boundary conditions related to the relative difficulty or task demands of semantic classification itself.

In developing a general paradigm to test this stage-specificity of incongruency encoding effects, we attempted to define a general set of inclusion/exclusion criteria for participant data, that (1) were well motivated theoretically, (2) served to exclude likely unreliable data while including as much data as possible, and (3) were independent of our primary memory measures of encoding difficulty. Considering that we are interested in the memory differences produced by difficulty manipulations at study, we excluded participants (1) with overall poor task performance at study (less than 75% correct in any condition), suggesting they were not performing the task adequately; and (2) who showed substantially reversed difficulty/priming effects at study (more than a 50 ms priming benefit for incongruent versus congruent primes), where we cannot be sure that our difficulty manipulation is actually making the task more difficult for those participants. Thus, our data reported here represent study congruency effects on later memory for participants (1) with reasonable study performance and (2) who were influenced as expected by difficulty manipulations at study.

## Experiment 1

For our first experiment, we used backward compatibility effect (BCE) response priming in the psychological refractory period (PRP) paradigm, to produce a response priming manipulation on a primary task (Task 1 of the dual-task PRP pair). In a typical PRP paradigm, participants are presented with two stimuli separated by a variable stimulus onset asynchrony (SOA), and respond to each stimulus in turn according to its own task set rules. We did not use this design to implement a typical dual-task difficulty manipulation where the degree of difficulty depends on single- versus dual-task performance, which has been generally shown not to produce difficulty benefits for memory (e.g., Craik et al., 1996; Gaspelin et al., 2013). Instead, here participants always performed a dual task. Our difficulty manipulation was within Task 1, where response congruency with automatically activated Task 2 response information provides the relative difficulty for Task 1 performance.

The backward compatibility effect is well studied, and is thought to reflect automatic stimulus-response translation and activation of Task 2 response representations, prior to any deliberate performance of Task 2 on a given trial, in parallel with attended Task 1 performance (Hommel, 1998; Hommel and Eglau, 2002; Watter and Logan, 2006; Ellenbogen and Meiran, 2008; Giammarco et al., 2016). This automatically generated Task 2 response information is observed to prime Task 1 RT, with converging evidence suggesting direct priming of the concurrent Task 1 response selection stage (Thomson et al., 2015). Using a PRP paradigm with two semantically unrelated tasks (here, a size categorization task on words for Task 1, and a shape classification task for Task 2) that both used the same pair of response keys enabled us to manipulate response congruency priming on the Task 1 response selection stage, without any priming of Task 1 semantic information.

Recent findings by Krebs et al. (2015) and Rosner et al. (2015a) suggest that enhanced demand for cognitive control (elicited through incongruent prime stimuli) should lead to better later memory. Chiu and Egner’s (2015) findings suggest that this should be the case if the control demand draws selective attention to the task and stimulus processing at hand, rather than divert this processing away to a secondary task (or in their case, a focus on withholding a response). In our experiment here, the BCE priming of Task 1 response selection occurs while participants are selectively and deliberately focused on performing Task 1 (*via* automaticity of directly activating Task 2 response information in the presence of the Task 2 stimulus, before participants change their focus of attention to deliberately perform Task 2). Importantly, our difficulty manipulation here maintains focus on the primary task, much like congruency manipulations of Krebs et al. (2015) and Rosner et al. (2015a), though our manipulations are selectively targeting response selection.

In addition to potential incongruency priming effects, presenting Task 1 and Task 2 stimuli at varying SOAs allowed us to independently assess a more general effect of divided attention on later memory. Dual-task interference is typically observed as a general divided attention or distraction effect on primary task performance when a distractor stimulus or task is present. As such, we might predict a similar general distraction effect on memory for stimuli presented at short versus long SOAs, separate to our primary congruency manipulations – that is, a distraction effect when a prime appears close in time with the primary stimulus, overlapping with much of attended Task 1 processing, versus when the prime appears toward the end of (or even after) Task 1 performance.

With respect to our principal focus on congruency/conflict effects, we predict that despite enhanced cognitive control demands with incongruent response priming, we should not observe incidental memory encoding benefits under higher conflict conditions here, as findings from Krebs et al. (2015) and Rosner et al. (2015a) might predict. We predict that priming conflict at response selection should focus selective attention and cognitive control on resolving response conflict, and as such there should be no enhanced processing of central stimulus representations under high-conflict conditions, and hence no incongruency memory benefit. Only in our later experiments, where difficulty/congruency manipulations draw selective attention and cognitive control to increase focus on central stimulus representations, should we observe incidental memory benefits with incongruent priming conditions.

### Method

#### Participants

Twenty first-year McMaster University students participated in this experiment for course credit. Informed written consent was obtained and McMaster’s Research Ethics Board approved the study. All participants reported normal color vision and normal or corrected-to-normal visual acuity, and spoke English fluently. Data from one participant were excluded due to low study task accuracy (<75% correct), and data from an additional two participants were eliminated due to substantially reversed (>50 ms) response priming effects on Task 1 RT during study task performance. Data from one other participant were lost due to unrelated technical issues, leaving 16 participants for reported data analysis. Our data collection employed a stopping rule of 20 or more participants, assessed at the end of each week of data collection. We based this *n* (number of participants) on typical numbers used in many PRP experiments to reliably study the backward compatibility effect (e.g., Hommel, 1998; Hommel and Eglau, 2002; Miller and Alderton, 2006).

#### Apparatus and Stimuli

All stimuli were presented on a standard Windows 7 PC and experiments were programmed in Presentation (v. 14, neurobs.com). Primary study and test stimuli were drawn from a list of 240 concrete nouns, all unambiguously classifiable along dimensions of animacy and size, with equal numbers of animate-small, animate-big, inanimate-small, and inanimate-big items. For the study/encoding phase, 160 of these words were presented once each as stimuli for Task 1 (S1) in a PRP paradigm. The other 80 words were used as foils (new items) in the subsequent memory test phase. To create counterbalanced study-test stimulus sets, we initially split the 240 words into three lists (A, B, C), balanced across lists for item category and the first letter of stimulus words. Participants saw stimuli from two of these three lists at study (e.g., A, B), and then were tested on stimuli from one of these two study lists plus the unseen third list (A, C). The six possible combinations of list arrangements were counterbalanced across participants in the experiment.

In the study/encoding phase, words were presented in white Arial font, sized to be 1.5 cm vertically on screen. Task 2 stimuli (S2) were one of four shape stimuli (star, diamond, circle, pentagon), presented in filled white, also 1.5 cm vertically. A pre-stimulus cue consisted of two rows of single dashes separated by spaces (“- -”), indicating a central position where stimuli would appear. Task 1 and 2 stimuli were presented in consistent positions centered on the screen, with S1 (word) always above S2 (shape), separated by approximately 0.75 cm gap. Participants sat at a viewing distance of approximately 60 cm from the screen. In the memory phase, single words were presented centrally in the same Arial font, at a larger size (approx. 2.5 cm vertically).

#### Procedure

**Figure 2:**
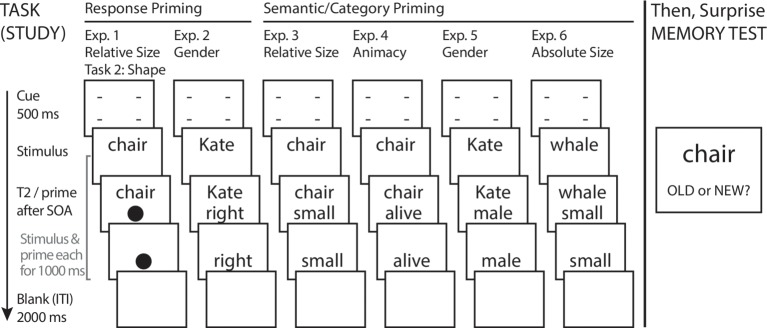
Task design and procedure for Experiments 1–6. Across experiments, participants classified stimuli along single dimensions of size, name gender, or animacy. An old/new recognition memory test followed each categorization task. T2 = Task 2 stimulus; ITI = Intertrial Interval.

The basic study design for this and subsequent experiments is shown in Figure 2. In the study/encoding phase, a single trial began with the cue presented for 500 ms. This was immediately replaced with S1 (word). After a variable SOA (150 or 700 ms, randomly varied), S2 (shape) was presented below S1 on the screen. Each stimulus was removed from the screen 1000 ms after presentation, giving a consistent exposure time for both stimuli across SOAs. Participants were instructed to prioritize Task 1, and not to move on to considering Task 2 until they had responded to Task 1. Response alternatives for both Task 1 and Task 2 were mapped to index and middle fingers of the right hand, using the “1” and “2” keys of the computer keyboard numeric keypad. Participants classified the referents of Task 1 word stimuli as bigger or smaller than the computer monitor, and classified shapes into (star or diamond) versus (circle or pentagon) sets. Response mapping for both tasks was counterbalanced across participants. An intertrial interval of 2000 ms (blank screen) separated the offset of S2 and presentation of the cue beginning a subsequent trial.

The study/encoding phase consisted of five experimental blocks, each with 32 trials, for a total of 160 trials. Trial information was pre-generated, with SOA and Task 2 shape iterated over Task 1 stimulus categories (with individual items pre-randomized within condition for every new participant), to ensure equal number of trials across conditions and randomize S1-S2-SOA groupings across participants. These condition-balanced and item-randomized trials were then presented to participants in random order. Using a separate 64 word stimulus set, an additional two blocks (32 practice stimuli each with randomized SOA and S2) were presented initially as practice, and not considered for memory test or analysis. Prior to data analysis, trials with Task 1 RT faster than 300 ms or slower than 2000 ms were excluded from analysis (less than 0.5% of all trials).

After the completion of the study/encoding phase, participants were given 2 min’ rest before proceeding to the surprise memory test. Stimuli consisted of 160 words – 80 old, shown during the encoding phase, and 80 new items, as outlined above. Participants were instructed to classify the words as “old” or “new” in relation to the encoding phase – whether they had seen that word during the encoding phase task. Stimuli were presented in randomized order, and remained on the screen until a response was made by pressing “Z” for old words or “/” for new words on the computer keyboard. A blank screen of 1000 ms separated response and the next memory stimulus. Trials were presented in blocks of 32 items, with short self-paced breaks in between.

### Results

#### Encoding Phase

**Figure 3:**
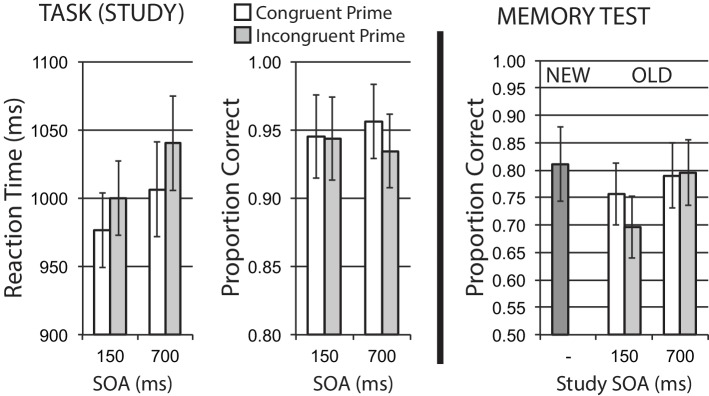
Size categorization task with response priming (Experiment 1). Response incongruency priming produces costs on categorization performance (left panel), but shows no evidence of an incongruency benefit for later memory (right panel). Error bars represent 95% CIs for congruent/incongruent mean paired differences, as a direct assessment of congruency effects.

Mean data for encoding phase Task 1 reaction time for correct trials, and Task 1 accuracy, are shown in the left half of Figure 3. A 2 × 2 repeated measures ANOVA on RT data that treated response congruency (congruent, incongruent) and SOA (150, 700 ms) as factors revealed a main effect of response congruency, *F*(1, 15) = 6.18, *p* = 0.025, $\eta_{p}^{2}$ = 0.29, with faster reaction times for congruent versus incongruent stimuli. There was no significant effect of SOA, *F*(1, 15) = 1.93, *p* = 0.185, $\eta_{p}^{2}$ = 0.11, and no interaction, *F* < 1. These data suggest that response incongruency presents a relative difficulty on Task 1 performance, replicating typical prior findings for the backward compatibility effect.

Task 1 mean accuracy at study was relatively high. Using the same ANOVA structure, no main effects were observed for response congruency or SOA, *Fs* < 1. While the interaction was not significant, *F*(1, 15) = 2.29, *p* = 0.151, $\eta_{p}^{2}$ = 0.13, the direction of response congruency difference observed at 700 ms SOA is toward reduced accuracy for incongruent trials, in accordance with a general difficulty manipulation.

Task 2 performance data are not directly relevant to our incongruency priming hypotheses or later memory data, but are presented here for completeness, and to confirm that our dual-task PRP design did indeed impose considerable dual-task costs on Task 1 performance. Mean data for Task 2 RT for correct Task 2 responses, and mean Task 2 accuracy, were analyzed for trials on which a correct Task 1 response was made (94.5% of all trials). Task 2 data were consistent with a typical dual-task PRP effect, with a substantial delay of Task 2 responding at short versus long SOA. Task 2 mean RT for correct trials was substantially slower at short SOA trials for both response-congruent (1219 ms) and response-incongruent (1235 ms) trials, compared to long SOA trials (834, 836 ms). This was reflected by a strong main effect of SOA, *F*(1, 15) = 1238.98, *p* < 0.001, $\eta_{p}^{2}$ = 0.99, with no main effect of congruency and no interaction, *Fs* < 1. Task 2 accuracy was numerically lower for response-congruent versus response-incongruent trials for both short SOA (90.2, 94.4%) and long SOA trials (90.7, 94.3%), but this effect of response congruency was not significant, *F*(1, 15) = 2.43, *p* = 0.140, $\eta_{p}^{2}$ = 0.14. There was no effect of SOA, and no interaction, *Fs* < 1. This numerical pattern of lower Task 2 accuracy for response-compatible trials is observed in other PRP studies exploring congruency effects between Task 1 and Task 2 (e.g., Watter and Logan, 2006), and is interpreted as a later “partial match” interference effect (Hommel, 2004, 2007) on Task 2, (e.g., a change in semantic information focus but with response information repeated), rather than any index of task difficulty during Task 1 performance.

#### Memory Phase

Figure 3 (right half) shows mean recognition memory performance (proportion correct) for old and new items at test, excluding the small number of items (per participant) incorrectly responded to at study. In this and subsequent experiments, old items were divided by the SOA and congruency priming conditions in which they were experienced in the prior study/encoding phase (the classification task). As the set of new items was not related to any of the particular study conditions, the calculated False Alarm (FA) rate for a given participant (incorrectly responding “old” to new items) was a single value – e.g., it is not possible in this design to calculate independent FA rates for congruent and incongruent conditions. As such, subtracting the same FA rate from congruent and incongruent Hit rates (correctly responding “old” to old items) for each participant would not alter statistical comparisons of our congruency effects for old items. For this and subsequent experiments, we reported and analyzed Hit rates for congruent and incongruent old items at respective study SOAs, and present proportion correct responses for the set of new items (Correct Reject responses) as a comparison.

Hit rates for old items served as the dependent variable in a 2 × 2 repeated measures ANOVA that treated stimulus study conditions of response congruency and SOA as factors. The analysis revealed a significant main effect of SOA, *F*(1, 15) = 7.20, *p* = 0.017, $\eta_{p}^{2}$ = 0.32, but no significant main effect of congruency *F*(1, 15) = 2.71, *p* = 0.121, $\eta_{p}^{2}$ = 0.15, with a non-significant interaction, *F*(1, 15) = 2.34, *p* = 0.147, $\eta_{p}^{2}$ = 0.14. Given the BCE response congruency manipulation at study is typically observed to influence performance maximally at short SOAs, we examined memory performance separately based on study SOA. A significant congruency effect on memory was observed for stimuli presented at the 150 ms SOA, *t*(15) = 2.27, *p* = 0.038, where congruent stimuli were better remembered than incongruent stimuli. Memory performance at the 700 ms SOA was extremely similar for congruency conditions, *t*(15) = −0.18, *p* = 0.856. The memory benefit for congruently primed stimuli at short SOA observed here is the opposite of an incongruency/conflict benefit on memory.

### Discussion

Experiment 1 imposed a response congruency priming manipulation on a size classification task, using the backward response compatibility effect from a semantically unrelated shape classification task within a PRP paradigm. Study task performance was in keeping with a relative difficulty effect on Task 1 PRP performance for response-incongruent trials. We observed a subsequent memory benefit for congruently primed stimuli at short SOA, compared to incongruently primed trials – memory was better for relatively lower conflict (response congruent) trials, with relatively worse memory for stimuli presented with a response-incongruent Task 2 stimulus. These results are consistent with the typical pattern of divided attention costs on memory (e.g., Craik et al., 1996). These results do not support a general account of task-focused cognitive control demand, where increased selective attention on incongruent trials improves incidental encoding (e.g., Krebs et al., 2015; Rosner et al., 2015a). This does not at all suggest that findings from Krebs et al. and Rosner et al. are incorrect, but suggests that a more processing stage-specific view of cognitive control demand may be required.

We note that in this first experiment, we also observed a more general dual task or divided attention influence of study task conditions on memory, independent of congruency condition. The significant main effect of study SOA on “old” recognition performance shows a 6.7% benefit for stimuli presented at 700 ms SOA compared to stimuli at 150 ms SOA. This effect is straightforwardly interpretable as a general divided attention or distraction effect, where having any prime presented close in time with the primary stimulus has a negative effect on incidental encoding, compared to primes presented half a second later, allowing more of the time course of Task 1 processing (most critically we presume, central representation of stimulus information) to be completed before potential distraction from the prime. In the present experiment, the size of this distraction difference on memory, due to simple overlap in time course (the effect of study SOA on memory) was comparable to the effect of congruency prime information at the short SOA (in fact, numerically larger). The observation of this kind of general divided attention/distraction effect with study SOA, independent of prime congruency relationships, is a useful manipulation check, and increases our confidence in our finding of no incongruency benefit to memory, given we can show our manipulation is sensitive to other kinds of similar attentional/control encoding effects on later memory. We anticipate this general divided attention influence of study SOA on memory in subsequent experiments, where it should also serve as a useful manipulation check for potential incongruency memory effects.

## Experiment 2

Experiment 2 aimed to replicate the basic response incongruency priming findings from Experiment 1 – an incongruency cost on initial study task performance suggesting greater difficulty, but no benefit of this difficulty at later memory test – using a single-task priming design akin to those used for the semantic priming experiments in the rest of the current paper. Krebs et al. (2015) observed an incongruency encoding benefit with memory for face stimuli with a gender classification task, with congruent versus incongruent word primes (“male” versus “female”). We adapted this idea to use typical male and female names as primary task stimuli in a name gender classification task, to push an interpretation of a potential encoding effect more strongly toward enhancement of central semantic representations rather than visual perceptual features.

We presented individual male and female names along with congruent and incongruent response primes (words “left” and “right”), and asked participants to identify the gender of the name, with responses assigned to left and right keys, while ignoring the prime words below. We again presented primes at short and long SOAs, but reduced the SOA durations in this experiment considering the generally faster time course of single-task versus dual-task performance.

### Method

#### Participants

Twenty-eight first-year McMaster University students participated in this experiment for course credit. Informed written consent was obtained and McMaster’s Research Ethics Board approved the study. All participants reported normal color vision and normal or corrected-to-normal visual acuity and spoke English fluently. Data from one participant were excluded due to low accuracy (<75%), and data from three participants were excluded due to substantially reversed RT priming effects during the encoding/study phase (>50ms incongruency benefit). A total of 24 participants were included for reported data analysis.

#### Apparatus, Stimuli, and Procedure

Methods were identical to Experiment 1 with the following exceptions. We presented name stimuli as for Task 1 in the dual-task PRP paradigm from Experiment 1, with response prime stimuli (words “right” or “left”) presented below the Task 1 stimulus in place of the original Task 2 shape stimuli. Prime words were in the same white Arial font as primary task stimuli, at 1.5 cm vertically on screen. Primes appeared following Task 1 word stimuli at SOAs of 17 ms (one video frame at 60 Hz) or 600 ms.

The single task was a name gender classification task (i.e., “Is this a male or a female name?”). Participants responded using the “Z” and “/” keys on the computer keyboard with left and right index fingers, with male/female category alternatives mapped to left and right keys, counterbalanced across participants. A final set of 240 typical Western/Anglophone names (120 female, 120 male) that we thought our participant pool would be familiar with, and that were not gender ambiguous (e.g., “Alex”), were compiled and reviewed by several independent raters (from an originally larger list). Three 80 item lists, each with 40 male and 40 female names, balanced across lists for first letter of names, were used to create six counterbalanced sets of study-test materials, following the same procedures as described in Experiment 1. This gave stimulus sets with 160 experimental trials at test, half of which were used at memory test with the remaining 80 items as new items. Sets of study trial conditions counterbalanced for prime congruency and SOA were created as described previously, and again presented in randomized order for each participant. Stimuli were presented in four blocks of 40 trials each. An additional 12 trials with separate name stimuli were initially presented as a practice block, and not considered for analysis or memory test. Prior to data analysis, trials with RT faster than 300 ms or slower than 1500 ms were excluded from analysis (less than 0.5% of all trials); in this and subsequent experiments, this lower threshold for too-slow RT performance was adopted considering expected performance for single-task versus dual-task demands. The memory task followed the same format as in Experiment 1, now with 160 total trials using name stimuli.

### Results

#### Encoding Phase

**Figure 4:**
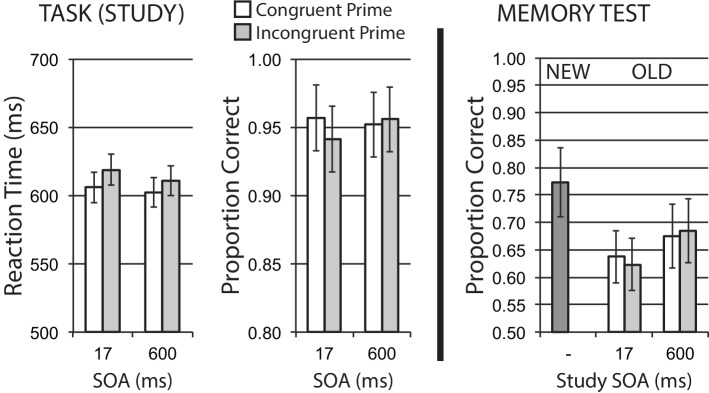
Name gender categorization task with response priming (Experiment 2). Response incongruency priming produces costs on categorization performance (left panel), but shows no evidence of an incongruency benefit for later memory (right panel). Error bars represent 95% CIs for congruent/incongruent mean paired differences, as a direct assessment of congruency effects.

Data for encoding phase mean RT for correct trials, and mean accuracy, are shown in the left half of Figure 4. A 2 × 2 repeated measures ANOVA revealed a main effect of congruency, *F*(1, 23) = 5.63, *p* = 0.026, $\eta_{p}^{2}$ = 0.20, with relatively slower RTs for incongruently primed trials reflecting the expected difficulty/congruency influence on initial task performance. The main effect of SOA was not significant, *F*(1, 23) = 1.20, *p* = 0.284, $\eta_{p}^{2}$ = 0.05, with no interaction, *F* < 1. Accuracy in the priming task was numerically worse for incongruent versus congruent trials at the short SOA, consistent with the difficulty manipulation, but the interaction of SOA and congruency was not significant, *F*(1, 23) = 2.23, *p* = 0.149, $\eta_{p}^{2}$ = 0.09, with no significant main effects, *Fs* < 1.

#### Memory Phase

Figure 4 (right half) shows mean recognition memory performance (proportion correct) for old and new items at test, excluding items (per participant) incorrectly responded to at study. A 2 × 2 repeated measures ANOVA for old items revealed a main effect of SOA, showing a general influence of divided attention with better subsequent memory performance from long versus short SOA trials, *F*(1, 23) = 5.91, *p* = 0.023, $\eta_{p}^{2}$ = 0.204. There was no significant effect of congruency, and no interaction, *Fs* < 1.

### Discussion

Experiment 2 used a single-task response priming design, with a gender name categorization task. As in Experiment 1, there was an influence of study SOA on later memory, reflecting a general divided attention/distraction effect for primes presented close in time to the primary task at short SOA, independent of prime congruency relationships. While there was clear evidence of response incongruency priming impacting classification task performance, there was no evidence of this encoding incongruency effect on later memory. These findings provide a conceptual replication of Experiment 1 with a single task design, again showing that incongruency/difficulty manipulations targeting response selection do not produce a related benefit in later memory. We again observe this lack of incongruency effect on memory while we are able to directly measure a separate encoding effect of divided attention/distraction (SOA) effect on memory, increasing confidence in our interpretation of this lack of an observable incongruency memory benefit.

## Experiment 3

Experiments 1 and 2 showed that when a targeted congruency/difficulty manipulation at the response selection stage produced increased cognitive control demand with incongruent primes, there was no benefit to later memory. For the rest of the present paper, we aimed to directly demonstrate how semantic incongruency priming in these same situations would produce encoding difficulty memory benefits where response priming had not. In this situation, we predict semantic processing conflict will draw selective attention and cognitive control processes to focus on central semantic and associative representations of the to-be-tested task stimuli, producing better memory encoding compared to congruently primed stimuli.

The aim of Experiment 3 was to begin to use the same primary tasks in our response priming experiments, now instead with a semantic congruency prime. For this experiment, we used the same size classification task as in Experiment 1, now as a single task with no Task 2. We presented semantic category primes (the words “BIG” and “small”) at short and long SOAs, in place of the Task 2 stimuli for Experiment 1, producing a semantic category congruency priming task, and again assessed later recognition memory. We reverted to the slightly longer SOAs used in Experiment 1, to allow a more direct comparison between response and semantic priming outcomes with the same primary size classification task. In addition, we aimed to double our sample size for this and subsequent experiments, adopting a stopping rule of 40 or more participants, assessed at the end of each week’s data collection.

### Method

#### Participants

Forty-six first-year McMaster University students participated in this experiment for course credit. Informed written consent was obtained and McMaster’s Research Ethics Board approved the study. All participants reported normal color vision and normal or corrected-to-normal visual acuity and spoke English fluently. Data from one participant were excluded due to low encoding phase accuracy (<75%). Data from four more participants were eliminated due to large reversed RT priming effects during the encoding/study phase (>50 ms incongruency benefit). A total of 41 participants were included for reported data analysis.

#### Apparatus, Stimuli, and Procedure

Methods were identical to Experiment 1 with the following exceptions. We adapted Task 1 from the dual-task PRP paradigm from Experiment 1 to a single task, with category prime stimuli (words “BIG” or “small”) presented below the Task 1 stimulus in place of the original S2 shape stimuli. Prime words were in the same white Arial font as primary task stimuli, at 1.5 cm vertically on screen. Participants responded to the size categorization task with left and right index fingers using the “Z” and “/” keys on the computer keyboard, with big/small response mappings counterbalanced across participants. We presented the same number of study/encoding trials, followed by the same memory test as in Experiment 1.

### Results

#### Encoding Phase

**Figure 5:**
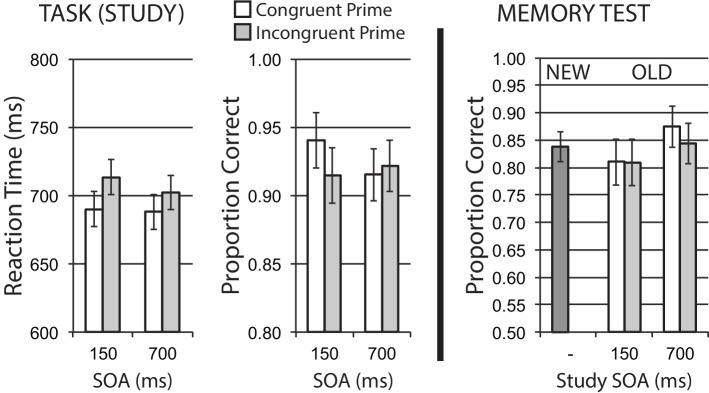
Size categorization task with semantic priming (Experiment 3). Semantic incongruency priming produces costs on categorization performance (left panel), but unexpectedly shows no evidence of an incongruency benefit for later memory (right panel). Error bars represent 95% CIs for congruent/incongruent mean paired differences, as a direct assessment of congruency effects.

Data for encoding phase mean RT for correct trials, and mean accuracy, are shown in the left half of Figure 5. A 2 × 2 repeated measures ANOVA revealed a strong main effect of congruency, *F*(1, 40) = 19.60, *p* < 0.001, $\eta_{p}^{2}$ = 0.33, with relatively slower RTs for incongruently primed trials reflecting the expected difficulty/congruency influence on initial task performance. The main effect of SOA was not significant, *F*(1, 40) = 2.01, *p* = 0.156, $\eta_{p}^{2}$ = 0.05, with no interaction, *F* < 1.

Accuracy in the priming task appeared to be relatively worse for incongruent versus congruent trials at the short SOA, with a significant interaction of congruency and SOA, *F*(1, 40) = 5.38, *p* = 0.026, $\eta_{p}^{2}$ = 0.12, but no significant main effects of congruency, *F*(1, 40) = 2.04, *p* = 0.161, $\eta_{p}^{2}$ = 0.5, or SOA, *F* < 1. Individual assessment of congruency effects at separate SOAs showed a significant accuracy benefit for congruent trials at the short SOA, *t*(40) = 2.57, *p* = 0.014, with no apparent difference at the long SOA, *t*(40) = −0.70, *p* = 0.491. These accuracy results align with RT results suggesting that incongruent primes create additional processing difficulty in task performance, as expected.

#### Memory Phase

Figure 5 (right half) shows mean recognition memory performance (proportion correct) for old and new items at test, excluding items (per participant) incorrectly responded to at study. A 2 × 2 repeated measures ANOVA for old items revealed a main effect of SOA, showing a general influence of divided attention/distraction with better subsequent memory performance from long SOA trials versus short SOA trials, *F*(1, 40) = 13.44, *p* < 0.001, $\eta_{p}^{2}$ = 0.25, as observed in previous experiments. Contrary to our predictions of an incongruency memory benefit with semantic priming, the main effect of congruency was not significant, *F*(1, 40) = 1.29, *p* = 0.263, with no significant interaction, *F*(1, 40) = 1.14, *p* = 0.292.

### Discussion

Despite our predictions, these data again suggest no evidence that increased attentional and cognitive control demands on incongruent trials improve incidental memory encoding, despite a clear and expected difficulty effect on initial task performance. We observed this lack of incongruency benefit on later memory despite using a semantic priming task, and again inconsistent with studies where increased selective attention on incongruent trials improves incidental encoding (e.g., Krebs et al., 2015; Rosner et al., 2015a). We note that we again observed a general effect of study SOA on later memory, consistent with a general effect of greater distraction by primes at the short SOA (independent of congruency condition), when primes overlap with a greater proportion of the time course of primary task processing, again encouraging our belief that we should be able to measure some degree of incongruency priming benefit were one to be present.

While we predicted that our response priming difficulty manipulations in Experiments 1 and 2 would not produce such memory effects, we were initially quite surprised by the present result, given our strong prediction that we should find an increase in incidental encoding here with semantic incongruency priming. Despite these results from Experiment 3, we still predict that a semantic congruency manipulation that elicits cognitive control and selective attention *via* incongruency conflict should enhance later memory, if those control processes enhance representations of the meaning of the to-be-tested stimuli. We address the particular issue of these unexpected semantic priming results with an additional clarifying test in Experiment 6. In the meantime, we employed several other semantic classification tasks with the same study design, to further explore these stage-specific predictions for congruency encoding effects.

## Experiment 4

The aim of Experiment 4 was to try to observe incongruency memory benefits with the same stimuli but a different categorization task from Experiment 3. The stimulus set used for the size classification task in Experiments 1 and 3 was composed of items counterbalanced on a second semantic dimension, animacy. It would be a powerful demonstration if we could show incongruency encoding effects using the same stimuli that had previously not shown such an effect, when classifying them along a different semantic dimension. Finding an incongruency encoding benefit with the same stimuli using an animacy classification task would suggest that something about our size task may have prevented us from producing an incongruency encoding effect in Experiment 3, and more importantly, might give us some insight into the nature of what kinds of priming are able to produce such effects.

We also reconsidered the timing of the SOAs we were using for these tasks. We had chosen our original SOAs of 150 and 700 ms for producing an optimal backward response compatibility effect with a dual-task PRP procedure in Experiment 1. We had persisted with these SOAs in Experiment 3 for consistency, but were concerned that they may be relatively slow for producing strong semantic category priming. As such, we returned to our shorter SOAs of 17 and 600 ms as in Experiment 2, to preserve the separate onsets of task stimulus and prime, but to introduce greater temporal overlap between prime and stimulus, and hopefully produce more effective semantic priming.

### Method

#### Participants

Forty-one first-year McMaster University students participated in this experiment for course credit. Informed written consent was obtained and McMaster’s Research Ethics Board approved the study. All participants reported normal color vision and normal or corrected-to-normal visual acuity and spoke English fluently. Data from one participant were excluded due to low accuracy (<75%) in the study/encoding phase, leaving 40 participants for reported data analysis.

#### Apparatus, Stimuli, and Procedure

Methods were identical to Experiment 3, aside from the following changes. We used the same stimulus sets as in Experiment 3, which were originally counterbalanced on both size (big, small) and animacy (alive, not alive) dimensions. We presented study/encoding trials in four blocks of 40 trials (rather than five blocks of 32 trials as previously), given the reduced trial length with shortened SOAs. Intertrial intervals were maintained at 2000 ms as before. Participants classified the referents of single word stimuli as either animate (alive) or inanimate (not alive). Primes were the words “animal” or “thing,” and were presented with counterbalancing and randomization procedures as previously described, at SOAs of 17 or 600 ms. A single practice block of 48 trials using a separate stimulus set (a subset of the prior 64 practice items) was presented at the beginning of the experiment, and was not considered for analysis or memory test. The memory test was the same as in Experiment 3.

### Results

#### Encoding Phase

**Figure 6:**
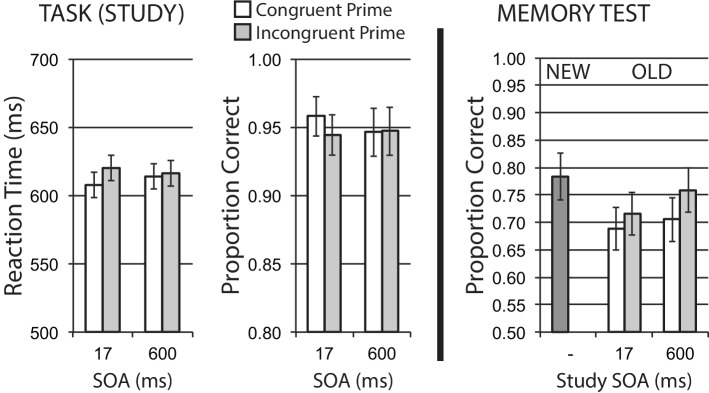
Animacy categorization task with semantic priming (Experiment 4). Semantic incongruency priming produces costs on categorization performance (left panel), and also produces an incongruency benefit for later memory (right panel). Error bars represent 95% CIs for congruent/incongruent mean paired differences, as a direct assessment of congruency effects.

Data for encoding phase mean RT for correct trials, and mean accuracy, are shown in the left half of Figure 6. A 2 × 2 repeated measures ANOVA of RT revealed a significant main effect of congruency, *F*(1, 39) = 4.53, *p* = 0.040, $\eta_{p}^{2}$ = 0.10, with no significant effect of SOA, *F* < 1, and a marginal interaction, *F*(1, 39) = 2.86, *p* = 0.098, $\eta_{p}^{2}$ = 0.07. These RT data suggest that the congruency manipulation was producing an expected difficulty effect on task performance.

Accuracy data showed no significant main effect for congruency, *F*(1, 39) = 1.71, *p* = 0.199, no main effect of SOA, *F* < 1, and no interaction, *F*(1, 39) = 1.26, *p* = 0.269. The direction of observed numerical differences in congruency conditions at the short SOA are consistent with RT data and the expected difficulty manipulation.

#### Memory Phase

Figure 6 (right half) shows mean recognition memory performance (proportion correct) for old and new items at test, excluding items (per participant) incorrectly responded to at study. A 2 × 2 repeated measures ANOVA for old item data revealed a strong main effect of congruency, *F*(1, 39) = 8.32, *p* = 0.006, $\eta_{p}^{2}$ = 0.18, with incongruently primed stimuli at study showing relatively better memory performance. This finding represents a substantial incongruency encoding benefit. There was no significant effect of SOA, *F*(1, 39) = 2.74, *p* = 0.106, $\eta_{p}^{2}$ = 0.07, and no significant interaction, *F* < 1.

### Discussion

Using the same concrete noun stimuli as in Experiment 3, but having participants classify items on the basis of animacy instead of size, we observed a substantial incongruency encoding benefit on later memory using semantic category primes for animacy information. These findings are consistent with the results from Krebs et al. (2015) and Rosner et al. (2015a), where greater incongruency or conflict for study items produced better later memory. While we did not observe a significant general divided attention/distraction effect of SOA as in previous experiments, data here were numerically consistent with this pattern. This is the first experiment in this paper where we observe our predicted incongruency encoding benefit for semantic incongruency priming. To generalize and extend this effect, we conducted a conceptual replication of this semantic congruency priming experiment, using the name stimuli and gender classification task from Experiment 2, with semantic category primes instead of response primes.

## Experiment 5

The aim of Experiment 5 was to again investigate the influence of task difficulty targeted at the semantic stage of processing, using a different categorization task. We used the same name gender classification task from Experiment 2, now presenting the individual male and female name stimuli along with congruent and incongruent semantic category primes (words “male” and “female”) in place of previous response primes (words “left” and “right”). Continuing our attempt to find optimal SOA conditions for single-task semantic priming to observe reliable difficulty encoding effects on memory, we used a single 100 ms SOA for this experiment. This was an attempt to maintain a similar subjective perceptual separation of stimulus and prime as in our prior experiments, but with a more optimal time course to better produce temporal overlap and maximize semantic priming. We again predicted that we should observe a congruency encoding effect on later memory test, with better memory for stimuli originally presented with incongruent semantic category primes.

### Method

#### Participants

Fifty-two first-year McMaster University students participated in this experiment for course credit. Informed written consent was obtained and McMaster’s Research Ethics Board approved the study. All participants reported normal color vision and normal or corrected-to-normal visual acuity and spoke English fluently. Data from two participants were excluded due to low accuracy at study (<75%), leaving a total of 50 participants for reported data analysis.

#### Apparatus, Stimuli, and Procedure

Methods were identical to Experiment 2, aside from the following changes. The primary task was the same name gender classification task (i.e., “Is this a male or a female name?”). Primes were the words “male” and “female.” Stimulus lists were constructed and counterbalanced as described previously, but now with all trials presented with a constant 100 ms SOA.

### Results

#### Encoding Phase

**Figure 7:**
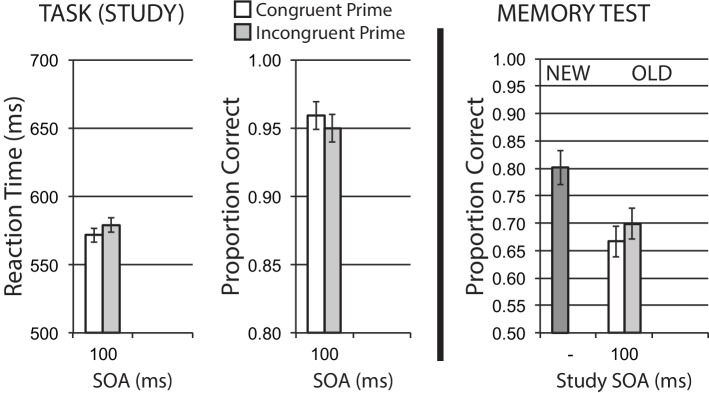
Name gender categorization task with semantic priming (Experiment 5). Semantic incongruency priming produces costs on categorization performance (left panel), and also produces an incongruency benefit for later memory (right panel). Error bars represent 95% CIs for congruent/incongruent mean paired differences, as a direct assessment of congruency effects.

Data for encoding phase mean RT for correct trials, and mean accuracy, are shown in the left half of Figure 7. A significant congruency priming effect was observed in RT, *t*(49) = 2.86, *p* = 0.006, suggesting that incongruent trials impose additional difficulty on task performance. Accuracy was numerically worse for incongruent trials, in line with this difficulty expectation, though this effect was only marginal, *t*(49) = 1.75, *p* = 0.086.

#### Memory Phase

Figure 7 (right half) shows mean recognition memory performance (proportion correct) for old and new items at test, excluding items (per participant) incorrectly responded to at study. A significant memory benefit for old stimuli with incongruent priming at study was observed, *t*(49) = 2.28, *p* = 0.027. These findings represent a clear memory benefit for items with semantic incongruency priming at study.

### Discussion

Experiment 5 showed a significant later memory benefit for stimuli initially experienced under more difficult incongruent semantic priming conditions. These data provide a conceptual replication of Experiment 4, and fit directly with our mechanistic prediction of increased cognitive control at semantic categorization to compensate for interference from an incongruent prime. These results are consistent with recent work (Krebs et al., 2015; Rosner et al., 2015a), where increased selective attention on incongruent trials leads to better later memory. Enhancing difficulty at the categorization stage of processing induces greater attentional control toward central semantic information of to-be-tested task stimuli, leading to better encoding and better later memory performance.

## Experiment 6

In Experiment 4 (animacy) and Experiment 5 (gender), stimuli in these different semantic classification tasks were better remembered when they were presented with incongruent versus congruent semantic primes. While Experiment 1 (size task) and Experiment 2 (gender task) showed no evidence of an incongruency benefit to memory with response priming, as predicted under our stage-specific encoding benefit model, there is still a question as to why Experiment 3 (size classification with semantic priming) did not show our predicted incongruency memory benefit, even though the very same stimuli did show an incongruency memory benefit when categorized on another semantic feature (animacy, in Experiment 4). One intriguing possibility that would fit this pattern of data may be the relative degree of automaticity involved with the classification task itself, or put another way, how essential or central the decision-relevant features for a given classification task are for the stimuli being classified.

If stimuli have strong associates or semantic features that are rapidly and automatically activated or retrieved under relevant classification task set rules, participants should typically have categorization-relevant information directly activated from semantic memory with little deliberation. In this situation, additional attentional control elicited through incongruent priming would focus selective attention on essential semantic information from the task stimulus.

On the other hand, if a category decision relies on additional comparative or evaluative work with the retrieved semantic contents from a stimulus in order to resolve a categorization decision, participants may end up performing a more controlled or algorithmic decision process no matter what the prime stimulus is. In this situation, the demands of the categorization task itself may act to elicit some greater degree of control in processing, or to simply involve a greater or richer extent of representation of stimulus information, for all trials.

A recent example that we suggest illustrates this kind of influence is studies of perceptual desirable difficulty using blurry versus clear word stimuli, by two different research groups (Yue et al., 2013; Rosner et al., 2015b). Both groups aimed to study essentially the same question – whether reading words that were presented under blurry or clear conditions would lead to subsequent better memory for the more difficult-to-read blurry items. Over many experiments, Yue et al. (2013) found a convincing absence of any encoding difficulty benefit on memory due to blurring of words, despite clear performance costs at study. Also over many experiments, Rosner et al. (2015b) found a consistent memory benefit for blurry versus clear words. Both studies were well conducted, and independently are quite convincing in their findings.

A critical difference determining these two robust and opposite outcomes was explored and verified in the final experiments of Rosner et al. (2015b) – the task performed at encoding. For experiments in Rosner et al. (2015b) showing a memory benefit of encoding difficulty, participants simply had to say the words – a relatively minimal task, and one where participants could rely substantially on automaticity in low-conflict (non-blurry) trials. In this task, the elicitation of more effortful and controlled processing in the presence of conflict/difficulty with blurry items leads to a memory benefit for those items, relative to the minimal-control conditions experienced for clear words.

In contrast, for Yue et al. (2013), the primary task required participants to make “judgments of learning” (JOL) for each clear or blurry word stimulus – that is, to explicitly evaluate how likely they thought it was that they would be able to remember that they had seen that word at study, on a later memory test. Rosner et al. (2015b) replicated this lack of memory effect when adding JOL responding to their procedures which had otherwise shown a difficulty memory benefit; they also discuss other studies in related memory literature that have similarly shown JOL procedures to disrupt other kinds of differential memory effects.

We suggest that the degree of engagement and effort required for this kind of more evaluative JOL task is substantially greater than simply reading or saying a word. With the JOL task, any potential differences in cognitive control elicitation due to processing difficulty or conflict from blurry words is unlikely to lead to substantial differences in central stimulus representations at study, as all stimuli are much more effortfully and completely represented because of the more demanding and evaluative task requirements themselves.

We propose that this kind of higher demand task situation was likely happening in our Experiment 3, where we did not find an incongruency memory benefit with semantic primes. In retrospect, our size classification task there is really a relative size comparison task, rather than a classification of essentially big and small items. Most items in this stimulus set were not canonically big or small (e.g., “elephant” or “flea”), and our explicit instructions were to compare many smaller or larger (but not canonically so) items relative to the size of the computer monitor. This is in contrast to the animacy task (Experiment 4), where with the exact same stimuli, we did find an incongruency memory benefit – here, animacy is an essential property of living but not non-living things, which we suggest participants have strong and relatively automatic memory access to within the task context of preparing to categorize items on the basis of animacy (we note that our animate items were all animals, not plants, and that our inanimate objects were either human-made items, e.g., “toaster,” or geologic features, e.g., “mountain”).

We predicted that we should be able to find an incongruency memory benefit for a size judgment task with semantic congruency primes (words “BIG” and “small”), if we used canonically big and small stimulus items that would allow participants to approach the task as a more direct semantic categorization task, rather than a more effortful evaluative task comparing each item to a reference object. We repeated the size classification task with semantic primes from Experiment 3, with a subset of canonically big and small items drawn from the larger stimulus set used in previous experiments, with instructions to simply judge items as typically big or small things.

### Method

#### Participants

Forty-four first-year McMaster University students participated in this experiment for course credit. Informed written consent was obtained and McMaster’s Research Ethics Board approved the study. All participants reported normal color vision and normal or corrected-to-normal visual acuity and spoke English fluently. Data from three participants were excluded due to low accuracy (<75%), and data from another three participants were eliminated due to substantially reversed priming effects (>50ms incongruency benefit), during the study/encoding phase. Another two participants were excluded due to an unrelated interruption of the experimental session and loss of data. A total of 36 participants were included for reported data analysis.

#### Apparatus, Stimuli, and Procedure

Methods were identical to Experiment 3, with the following exceptions. From our original set of 240 concrete nouns, we selected a subset of 96 words (half big, half small) that were canonically big and small items, excluding items where size was not a central semantic feature. These words were divided into three 32 item lists, with stimulus-test sets generated as described in previous experiments. For a given participant, this gave 64 items at study, with half of these items presented at test as “old” items, with the remaining 32 items as “new” items. Prime words were again “BIG” and “small.” We continued with the single SOA at 100 ms for this experiment. An initial practice block of eight trials presented a separate set of absolute big or small stimuli, which were not assessed or considered for the memory test. The memory test was the same as in previous experiments, with two blocks of 32 items each, in random order.

### Results

#### Encoding Phase

**Figure 8:**
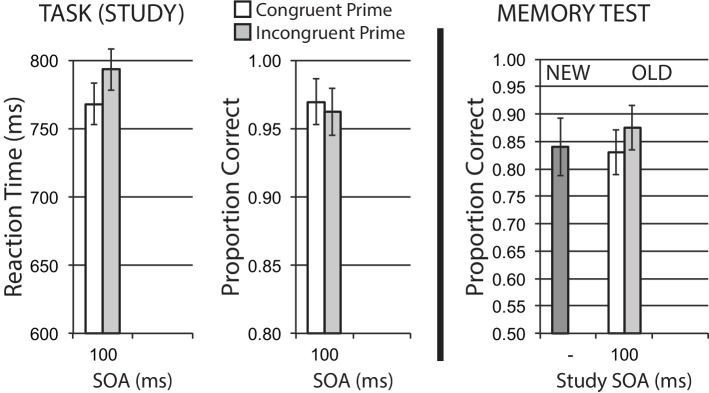
Size categorization task with semantic priming (Experiment 6), using canonical big/small stimuli. Semantic incongruency priming produces costs on categorization performance (left panel), and also produces an incongruency benefit for later memory (right panel). Error bars represent 95% CIs for congruent/incongruent mean paired differences, as a direct assessment of congruency effects.

Data for encoding phase mean RT for correct trials, and mean accuracy, are shown in the left half of Figure 8. A significant congruency priming effect was observed in RT, *t*(35) = 3.39, *p* = 0.002, suggesting that incongruent trials impose additional difficulty on task performance. Accuracy was numerically worse for incongruent trials, in line with this difficulty expectation, though this effect was not significant, *t*(35) = 0.90, *p* = 0.372.

#### Memory Phase

Figure 8 (right half) shows mean recognition memory performance (proportion correct) for old and new items at test, excluding items (per participant) incorrectly responded to at study. A significant memory benefit for old stimuli with incongruent priming at study was observed, *t*(35) = 2.23, *p* = 0.032. These findings represent another clear memory benefit from semantic incongruency priming.

### Discussion

In Experiment 6, we observed a convincing incongruency encoding benefit with semantic congruency priming in a size classification task, when we used canonically big or small stimulus items. This is in direct contrast to the lack of any incongruency memory benefit with the same big/small size classification task in Experiment 3, where most stimuli required a more relational comparison of size relative to a common middle-sized reference object.

While it is possible that in Experiment 3 we may have a subset of these canonical big/small stimuli that have a hidden encoding effect, we suspect that the more evaluative or relational processing required for the majority of stimuli likely induces this kind of processing as a general approach to the task for most trials. We conducted a small number of follow-up analyses on a subset of Experiment 3 canonical big/small stimuli, but did not find a comparable incongruency encoding benefit for memory there. For participants to take advantage of automaticity in categorization, they may require the more general task situation to support this. We suggest that this difference in primary task demands moderating incongruency memory effects and the similar task dependency of difficulty-related memory effects with blurry versus clear perceptual desirable difficulty described by Rosner et al. (2015b) are both examples of a broader limitation of task processing demands on encoding difficulty effects. We discuss these issues more in the General Discussion section, below.

## General Discussion

Several recent papers have suggested that the demand for increased selective attention and cognitive control on incongruent trials leads to better incidental encoding of task stimuli (e.g., Krebs et al., 2015; Rosner et al., 2015a). Similarly, if inhibitory control demands redirect the focus of selective attention away from stimulus processing, a relative memory difference can again be observed (Chiu and Egner, 2015). Our present findings are directly in line with all of these recent results, and present substantial additional detail about the stage-by-stage processing dependencies involved in producing such encoding effects on later memory.

We demonstrate that these incidental encoding effects on memory can be produced by semantic incongruency priming, when primes induce additional attention and control at a processing stage that is focused on the core meaning of to-be-tested task stimuli. We demonstrate these effects with animacy classification using concrete nouns (Experiment 4), with male/female name gender classification (Experiment 5), and with size classification using concrete nouns (Experiment 6). In all of these situations, categorization tasks targeted core semantic features and/or strong associate information of these classes of stimuli, i.e., gender for typical/traditional names, animacy for animals versus inanimate objects, and size for a set of canonically big or small items (e.g., “elephant” versus “flea”). We suggest that in all of these cases, the categorization task required minimal evaluative processing to determine the category decision. In the presence of an incongruent semantic prime, increased conflict/control would increase focus on the rapidly and automatically activated semantic and strong associate content of the item itself, resolving categorization conflict and also leading to relatively better encoding of item information.

In contrast, using size (Experiment 1) and name gender (Experiment 2) classification tasks, we show a lack of these incongruency encoding effects on memory when we prime response representations. In these cases, we suggest that additional attention and control are focused on central response selection processing, in order to resolve the conflict and competition induced by priming with incongruent response information. This diversion of cognitive control focus away from central representation of stimulus information with response priming predicts that later memory should not benefit from an increase in elicited cognitive control on incongruent/high-conflict trials, as we demonstrate. Our dissociation of conflict-related costs on task performance in general, versus the selectivity with which particular kinds of processing conflict at study will produce later memory benefits, is an important new finding for this broader literature.

An additional possibility is that with sufficiently strong control demands at response selection from incongruent response priming, attentional focus on semantic representations of stimulus information could be reduced or cut short, leading to relatively poorer encoding of stimulus semantic information and a relative cost on later memory. A strict interpretation of this mechanism would predict that this should occur only at shorter SOA, where response primes would generate conflicting response information relatively early in the time course of Task 1 to strongly influence response selection; in contrast, this effect should be much reduced at long SOAs where substantial amounts of semantic encoding could occur for both congruency types prior to the onset and influence of the response prime. Data from Experiment 1 (see Figure 3) show this precise pattern, with incongruency costs on memory selectively at the short 150 ms SOA, and no congruency effect at long SOA, even though initial task RT performance is similarly affected by incongruent response priming at both SOAs. It is possible that the backward compatibility response priming in the dual-task PRP design in Experiment 1 was a particularly potent method of Task 1 response selection priming, compared to the “left”/“right” word primes in the single-task Experiment 2.

In addition, and most strikingly, in Experiment 3 we showed that semantic congruency priming failed to produce an incongruency encoding memory benefit, despite using the same size categorization task and prime stimuli that produced an incongruency encoding benefit in Experiment 6, and the same word stimuli that produced an incongruency encoding benefit with animacy classification in Experiment 4. We suggest that the critical difference between Experiments 3 and 6 with the same size categorization task is the nature of the word stimuli we used, and subsequently the kind of categorization performance participants were required to perform because of this. We suggest that Experiment 3 required a more effortful relational or comparative assessment of size information from stimuli that mostly did not have big or small size as a central semantic feature or strong associate, and that this increased evaluative processing demand of the primary task itself lead to better central representation of stimulus information for all trials, minimizing any potential semantic incongruency memory effect.

This finding, along with similar findings of encoding difficulty effects being dependent on primary task demand (e.g., saying versus making judgments of learning on blurry versus clear words, and more general disruption of memory effects with judgment of learning tasks within the broader literature) as discussed by Rosner et al. (2015b), leads us to a second important consideration about likely mechanisms of incidental encoding difficulty effects on memory in general. Within general task designs that can be shown to elicit difficulty-related memory benefits, we have seen that added processing or evaluative demand in and of the primary task itself can abolish the memory benefit of greater task difficulty, despite this difficulty manipulation still imposing considerable costs in initial performance in all cases. The evaluative assessment in these tasks requires attentional control that benefits encoding of all items, independent of congruency or general task difficulty. This strongly suggests that in situations where we do observe conflict encoding benefits, the difference represents a relative cost to encoding in low-conflict conditions (involving relatively fluent performance with considerable support from automaticity), rather than a special enhancement to encoding under high-conflict conditions eliciting more controlled attentional processing.

Critically, we suggest that difficulty encoding effects represent a contrast between lesser-versus-normal control and attentional engagement, rather than a normal-versus-enhanced difference, where “normal” means the kind of engagement and processing that might be achieved if participants fully attended to and considered the stimulus with good, focused, endogenous top-down control. Put another way, difficulty encoding effects may be showing us that participants have relatively minimal semantic engagement with familiar stimuli and simple tasks under low-conflict conditions – interpreting difficulty memory benefits against this low-control baseline condition is valid and indeed revealing, but we suggest that the “benefit” to memory here is only a benefit with respect to an inherently encoding-poor situation. Inducing participants to focus more on the content or meaning of stimuli with different task demands (e.g., comparative size judgments, or judgments of learning) quickly equates memory for all stimuli, despite other differential difficulty manipulations. We agree with the idea that increased trial conflict or difficulty is likely to elicit increased cognitive control (e.g., Verguts and Notebaert, 2008; Krebs et al., 2015), but suggest that this may not additionally enhance encoding where stimulus information is already strongly attended to and represented by demands of the task itself. This interpretation is a pessimistic one with regard to the broader desirable difficulty and related cognition and education literatures – it suggests that making a task more engaging in itself is a better path to retention of content, and that these kinds of conflict encoding benefits, arguably a major focus of a wide array of perceptual desirable difficulty benefits, may only be beneficial against a backdrop of minimal engagement with the meaning of any task material.

A more stage- and process-specific approach to considering conflict effects on memory may also help us align other recent findings in this emerging literature. Several recent studies (Ortiz-Tudela et al., 2017, 2018) have shown what on the surface appears to be an opposite memory effect of incongruency for objects displayed in congruent versus incongruent background scene contexts – incongruent items were quicker to be identified and localized (though with more error) in a change detection task, but showed worse later memory compared to congruent items. The authors discuss their findings as being at odds with theories of conflict-elicited learning (Verguts and Notebaert, 2008, 2009), but compatible with more general principles such as desirable difficulty or depth of processing. We suggest that a task analysis of what processes elicit more or less cognitive control, and where that control is subsequently focused, is both consistent with presumed mechanisms and data from change detection, and also predicts the observed memory results.

In our own incongruency priming tasks here (and for Rosner et al., 2015a, and others), incongruent priming adds noise to the classification process (more information for the alternative incorrect category for a given stimulus), and participants are forced to employ a greater degree of top-down cognitive control in order to elicit adequate semantic feature/category information from the stimulus. More simply, our conflict condition makes participants do more high-level attentional work, and in our semantic priming conditions, this work is directly focused on the meaning or essential category information of the stimulus itself. In contrast, in the change blindness studies (Ortiz-Tudela et al., 2017, 2018), the incongruent condition provides a strong automatic (and presumably rapid, pre-volitional) cue, both that something does not match, and also possibly a spatial cue to where the contextually inappropriate object is in the scene. In this case, it is the congruent condition that requires more deliberate attentional work and controlled processing to find the changing object. The authors themselves describe essentially this in terms of “desirable difficulty” (Ortiz-Tudela et al., 2017). We would agree, and suggest that beyond a concept of general difficulty, elicitation of greater cognitive control that focuses processing on to-be-tested information should lead to better memory performance – the particular circumstances of the change detection task allow participants to do less deliberate, effortful controlled search (hence giving worse memory) due to the automaticity benefits of detection of contextual mismatch in visual scenes.

Finally, one potential limitation within our results is that while Experiment 1 used a response priming manipulation to show no incongruency memory effects, the primary task was the same relative size categorization task used in Experiment 3, where we similarly found no semantic incongruency memory effects. While this is a possible limitation in Experiment 1, our direct dissociation of memory effects between Experiments 2 and 5 (name gender classification with response priming versus semantic priming) provides an additional independent demonstration of our basic stage-specific findings on incongruency encoding benefits.

### Implications for “Desirable Difficulty” Effects

These selective attention-related encoding benefits may be highly relevant to the broader literature of desirable difficulty effects (Bjork and Bjork, 1992, 2011; Bjork, 1994), where difficulty experienced while processing an item (most commonly during retrieval) promotes better long-term memory. Well-documented examples of desirable difficulties include spaced practice (for a review, see Cepeda et al., 2006), interleaving study materials (Rohrer and Taylor, 2006; Kornell and Bjork, 2008), and test-enhanced learning (e.g., Roediger and Karpicke, 2006). In addition to these, processing difficulties during initial encoding, in the form of perceptual interference (Nairne, 1988; Hirshman and Mulligan, 1991), hard-to-read fonts (Diemand-Yauman et al., 2011), and inverted words (Sungkhasettee et al., 2011), have been shown to enhance retention. Processing difficulties such as these therefore appear to be “desirable” for learning.

However, not all processing difficulties are desirable for learning. Indeed, the broader history of experimental psychology has largely converged on a view that additional task difficulty tends to lead to worse performance, both in the moment and for later memory. For example, divided attention tasks are difficult, but typically impair memory for learned material (Baddeley et al., 1984; Craik et al., 1996; Mulligan, 1998; Fernandes and Moscovitch, 2000; Dudukovic et al., 2009; Gaspelin et al., 2013; but for an exception, see Kessler et al., 2014). The desirable difficulty framework established by Bjork and Bjork (1992, 2011) has been influential in steering researchers to consider situations where these apparently general costs might be avoided, or even reversed.

As Chiu and Egner (2015) suggest from their recent cognitive control manipulations that direct attention away from stimulus encoding (inhibitory control tasks) rather than toward it, it is reasonable to assume that in order for a difficulty manipulation to induce a memory benefit, the difficulty must increase selective attention to the to-be-remembered information. However, more than an attentional or control focus toward or away from a primary task, our present experiments show that this is an even more specific requirement. We suggest that the particular stage of processing that is the recipient of facilitation or conflict is likely to be a critical consideration for predicting whether a “desirable difficulty” effect on later memory will occur. Put another way, our results show that task difficulty in general does not improve memory, but instead will occur only in cases where the information to be encoded (to be assessed at later memory test) was the beneficial recipient of the enhanced attention and cognitive control required by the task.

The results of the current study suggest a stage-specific model of desirable difficulty in several ways, and may help to clarify other issues in this literature. Firstly, our study may contribute to continuing interpretation of relevant classic prior work, showing that more difficult encoding conditions produce memory benefits (e.g., Jacoby et al., 1976). Additionally, it might be argued that difficulty benefits on later memory may arise simply due to available time on task – that the additional time required to process and respond to high-conflict compared to low-conflict items directly produces additional encoding through longer exposure, leading to a memory benefit. Our experiments can address both of these issues. As can be seen in Experiments 1, 2, and 3, although conflict and extra time on task were present for incongruent versus congruent trials during primary task performance, no memory benefits were found. Enhanced memory is not apparent for all difficult selective attention-encoding conditions, but rather depends on the particular stage of processing at which this additional difficulty occurs.

### Conclusion

Taken together, our results suggest a highly stage-specific mechanism for producing conflict/control-related incidental encoding effects. We generally agree with accounts in the literature suggesting that conflict resolution involving top-down attention toward task-relevant information should facilitate memory for that information (e.g., Egner and Hirsch, 2005; Botvinick, 2007). Furthermore, our results are similar to those in recent studies showing memory effects from congruency priming tasks, where incongruent (higher conflict) items are better remembered relative to congruent (lower conflict) items (e.g., Krebs et al., 2015; Rosner et al., 2015a). We agree with these authors that an increase in selective attention due to increased cognitive control elicitation from incongruent stimuli likely provides a memory encoding benefit for incongruent versus congruent items in these cases. However, we show an important constraint on this kind of effect – that this encoding benefit is stage-specific, and only occurs when additional control is directed at a processing stage focused on the representation of to-be-tested information. In some cases, task demands will serve to focus this difference in processing conflict at a semantic representation stage, and we observe memory benefits in high-conflict situations. On the other hand, the variability of results in the broader desirable difficulty literature, not to mention that conflict-related desirable difficulty itself is still a somewhat novel and surprising effect within psychology’s long history of divided attention costs, suggests that “difficulty” simply being task- or stimulus-directed might not be sufficient for eliciting memory benefits.

Further, we suggest that difficulty/conflict encoding benefits are likely to be observed when demands of the task itself are relatively lower and allow a degree of automaticity in responding – if the task itself requires substantial evaluative work, additional attentional and cognitive control focus on stimulus information from stimulus-focused difficulty manipulations does not seem to further enhance memory encoding, even though it imposes a cost on initial task performance. In this sense, conflict/difficulty encoding benefits as a general class of effects might be limited both to (1) situations where cognitive control demand focuses processing on to-be-tested information, and also (2) situations where typical task engagement is relatively fluent, automatic, and encoding-poor, rather than cognitive control elicitation having some additional encoding benefit in all situations. It is important to note that this need not be a deliberate experimental manipulation of semantic congruency – the critical consideration is what information ends up as the focus of central attention, rather than the particular task manipulation used to achieve this. We suggest that many desirable difficulty effects where interference manipulations are purely perceptual will often still focus central attention on to-be-tested stimulus information. So long as those perceptual manipulations and related task requirements do not require too much effortful or evaluative work to access or represent relevant stimulus meaning, we would expect memory benefits from differential attentional encoding effects for more demanding perceptual conditions, against a background of relatively fluent and automatic performance in low-conflict conditions.

The present results should be investigated further from a desirable difficulty perspective. Further research should make it possible to make better predictions about how and where these kinds of processing conflict or desirable difficulty effects should occur, and that fundamental ideas and knowledge we already have within cognitive psychology might provide more guidance than we may have suspected. For now, these results provide evidence toward a stage-specific model that predicts when incongruency conflict in task performance should and should not lead to better incidental encoding of task stimuli.

## Ethics Statement

All procedures performed in studies involving human participants were in accordance with the ethical standards of the institutional and/or national research committee and with the 1964 Helsinki declaration and its later amendments or comparable ethical standards. Informed consent was obtained from all participants included in the study.

## Author Contributions

MP and SW both played a significant role in all aspects of this project including the experimental design, entry, analysis, interpretation of data, the drafting and revising of this manuscript, and final approval for publication. KH was involved with the experimental design, interpretation of data, and the revising of this manuscript. ST was involved with the experimental design, analysis, interpretation of data, and the revising of this manuscript.

### Conflict of Interest Statement

The authors declare that the research was conducted in the absence of any commercial or financial relationships that could be construed as a potential conflict of interest.
